# Motion Simulation of Ionic Liquid Gel Soft Actuators Based on CPG Control

**DOI:** 10.1155/2019/8256723

**Published:** 2019-02-26

**Authors:** Chenghong Zhang, Bin He, An Ding, Shoulin Xu, Zhipeng Wang, Yanmin Zhou

**Affiliations:** College of Electronics and Information Engineering, Tongji University, No. 4800 Caoan Road, Shanghai 201804, China

## Abstract

The ionic liquid gel (ILG), a new type of soft actuator material, is a mixture of 1-butyl-3-methylimidazolium tetrafluoroborate (BMIMBF_4_), hydroxyethyl methacrylate (HEMA), diethoxyacetophenone (DEAP), and ZrO_2_ polymerized into a gel state under ultraviolet (UV) light irradiation. The soft actuator structure consists of a layer of ionic liquid polymer gel sandwiched between two layers of activated carbon capped with gold foil. The volume of the cationic BMIM^+^ in the ionic liquid BMIMBF_4_ is much larger than that of the anionic BF_4_^−^. When voltages are applied to both sides of the actuator, the anions and cations move toward the anode and cathode of the electrode, respectively, under the electric field. The volume of the ILG cathode side therefore expands, and the volume of the ILG anode side shrinks, hence bending the entire actuator toward the anode side. The Ogden model was selected as the hyperelastic constitutive model to study the mechanical properties of the ILG by nonlinear analysis. As the ILG is an ideal material for the preparation of a supercapacitor, the equivalent circuit of the ILG can be modeled by the supercapacitor theory to identify the transfer function of the soft actuator. The central pattern generator (CPG) control is widely used in the area of biology, and CPGs based on bioinspired control methods have attracted great attention from researchers worldwide. After the continuum soft actuator is discretized, the CPG-based bioinspired method can be used to control the soft robot drivers. According to the simulation analysis results, the soft actuator can be smooth enough to reach the specified location.

## 1. Introduction

Due to the large difference in structure between the soft robot and the traditional robot, the material used to manufacture a robot for traditional applications is quite different from the material used to manufacture a bionic robot. In the past few decades, new lightweight, high-performance materials have attracted the attention of soft robot researchers worldwide. The electroactive polymer (EAP) has been proven to be a proper smart material that meets the requirements of soft robot design. The application of EAP materials has become a popular topic this year. Bionic robots and equipment using materials such as soft actuators have been produced [1, 2]. Because the soft robot offers strong adaptability and low pressure impedance, it has wide application prospects in biology, medicine, and agriculture. Electrochemical actuators have been developed quickly over the past few decades due to their desired mechanical properties in intelligent robots [3–5]. Because ionic liquid gels (ILGs) offer chemical stability, thermal stability, and simple ion transport, they are suitable for the production of soft robot actuators [6–8].

Noncovalent interactions allow supramolecular gel materials to have a very high mechanical strength and excellent self-healing ability. It has been demonstrated and well documented that ZrO_2_ can improve the electrochemical behavior of ionic liquids and the mechanical strength of ionic polymers in supramolecular nanocomposites [9].

The application of a bioinspired control method based on the central pattern generator (CPG) of neural oscillators to generate rhythmic motion has attracted the attention of some researchers [10–12]. The rhythmic movement of animals is achieved by the interaction between the rhythm signal generated from the CPG and the dynamics of the musculoskeletal system [13–15]. Nonlinear oscillators have been widely used to model rhythmic motion-generating CPGs for robot control.

**Figure 1:**
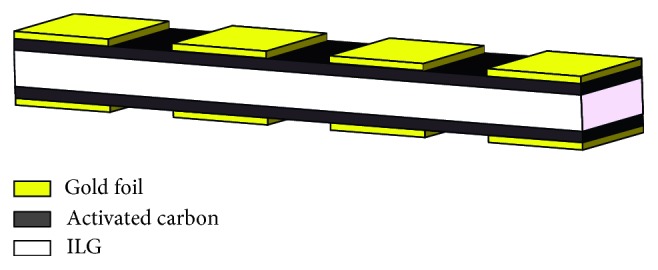
The 5-layer structure of the flexible actuator.

In this paper, a new type of ILG soft actuator is demonstrated. The soft actuator is composed of a middle layer of ionic liquid polymer gel sandwiched between two layers of activated carbon capped with gold foil, as illustrated in Figure 1. The ILG of the soft actuator was prepared, and its mechanical properties were described. The bending deformation principle of the soft actuator is discussed. The soft actuator is discretized to meet the conditions of the CPG control method, of which the effectiveness has been proven by numerical simulations. This paper provides a detailed theoretical analysis of ILG soft actuators based on the aspects of design, material, control, etc. This work has introduced a new research direction for the development of soft actuators and will contribute to the development of soft robots.

## 2. Structure Design of a Soft Robot Actuator

The new type of flexible actuator structure shown in Figure 1 is similar to the traditional EAP actuator. The white area is the ionic liquid polymer gel, which is the structural body of the actuator. The black sides are the activated carbon layers, which adsorb the anions and cations of the ionic liquid, respectively. The outermost yellow area is the gold foil, and each side of the gold foil is cut into 4 segments, each of which is connected to a power source.

The activated carbon layer is used to adsorb the anions and cations in the ionic liquid polymer gel, the gold foil layer is used as the electrode, and a wire is connected on the outside of the gold foil to the power source.

## 3. Ionic Liquid Gel

### 3.1. Ionic Liquid Gel Properties

In the experiment, the ILGs were composed of 1-butyl-3-methylimidazolium tetrafluoroborate (BMIMBF_4_), hydroxyethyl methacrylate (HEMA), ZrO_2_ nanoparticles, and 2,2- diethoxyacetophenone (DEAP), with masses of 890 mg, 68.6 mg, 30 mg, and 1.4 mg, respectively. A mixed solution of the ILGs was prepared in the ratio mentioned above, and then the mixed solution was stirred in a magnetic stirrer for 60 minutes or longer to form a suspension. The suspension was then placed under ultrahigh-intensity UV light generated by an ML-3500C Maxima-type cold light source and then polymerized to the gel state. The ML-3500C Maxima lamp has an ultraviolet intensity of 90,000 *μ*Ws/cm^2^ (15″/380 mm distance) and a wavelength of 365 nm.

**Figure 2:**
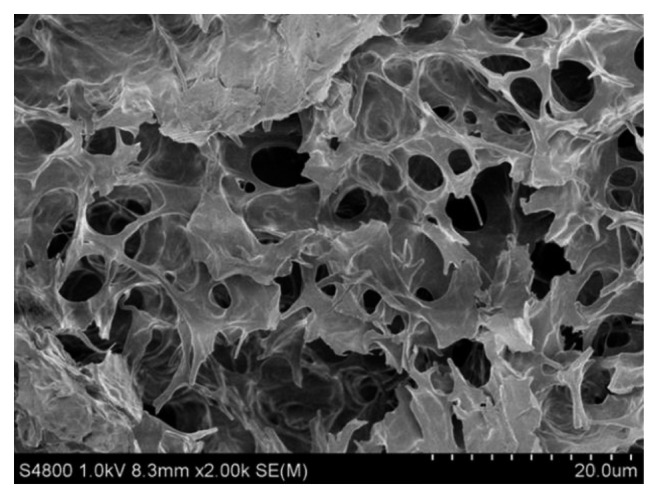
SEM image of a freeze-dried BMIMBF_4_-based gel after the ionic liquid was replaced with water.

The morphological scanning analysis results of freeze-dried samples was obtained by scanning electron microscopy (SEM) and showed that the existence of porous microstructures in ionic gels is common. The internal liquid of the ILG was replaced with distilled water. After the freeze-drying treatment, a Hitachi S4800-type high-resolution field emission SEM was used to scan the section.  Figure 2 shows the typical 3D porous structure of the ionic liquid carrier HEMA with a magnification of 5000. The polymerization of HEMA in the solution occurs under UV irradiation, and the polymer matrix is crosslinked to form a porous network structure [15]. The crosslinked matrix forms a 3D framework, offering good mechanical strength and self-repair properties.

**Figure 3:**
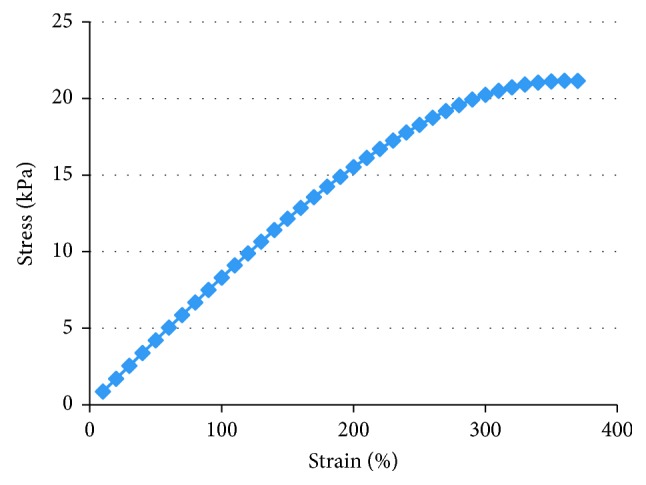
Stress-strain curve of ionic liquid polymer gels.

The BMIMBF_4_-based ionic gel offers a high level of hyperelastic toughness, with tensile deformation reaching as high as 360%, as shown in Figure 3. The average tensile strength (Young's modulus) of the material obtained from the tensile stress-strain curve is 7.6 kPa. The tensile tests show that the tensile properties of the gel increase with increasing ZrO_2_ content. An increase in the amount of ZrO_2_ will result in a larger number of crosslinking sites and higher conversion rates for HEMA, which can improve the final mechanical properties of the ILGs. Based on the tensile deformation and tensile strength data above, the optimized amount of ZrO_2_ is selected as 3 wt.%.

### 3.2. Driving Principle Analysis

The volumes of cationic BMIM^+^ and anionic BF_4_^−^ in the ionic liquid BMIMBF_4_ are very different. The volume of cationic BMIM^+^ is much larger than that of anionic BF_4_^−^. If voltages are applied on both sides of the actuator, the anions and cations will move toward the anode and cathode of the electrodes, respectively, under the electric field. The ions penetrate through the contact boundary of the ionic liquid polymer gel layer and the activated carbon layer; afterwards, the ions are strongly adsorbed by the activated carbon powder and accumulate in the activated carbon layer.

**Figure 4:**
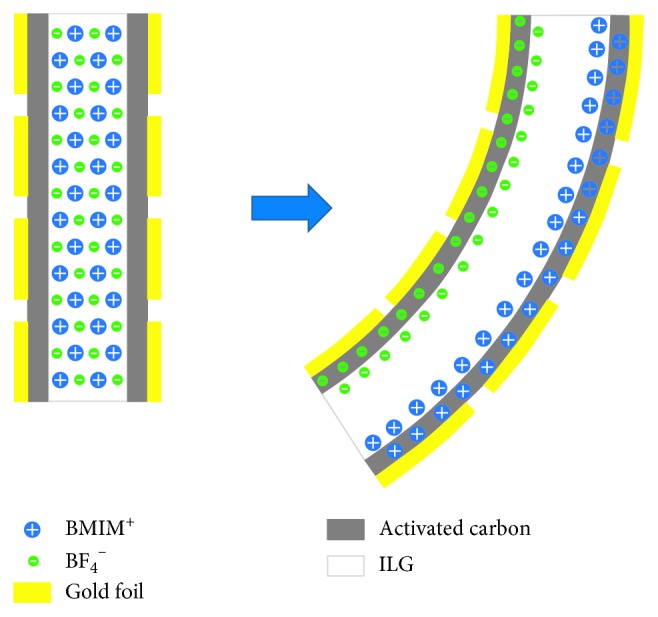
The working principle of the actuator.

The external voltage will lead to the accumulation of the opposite charges of the two electrodes. The charges will interact with the immobilized anions in the bulk polymer, as shown in Figure 4. The interaction will attract one electrode and repel the other.

When the ion motion reaches a stable state, due to the difference between the volumes of the anions and the cations, the volume of the ILG cathode side thus expands, and the volume of the ILG anode side shrinks. The entire actuator is therefore bent toward the anode side, as shown in Figure 4.

## 4. Hyperelastic

### 4.1. Hyperelastic Stress

**Figure 5:**
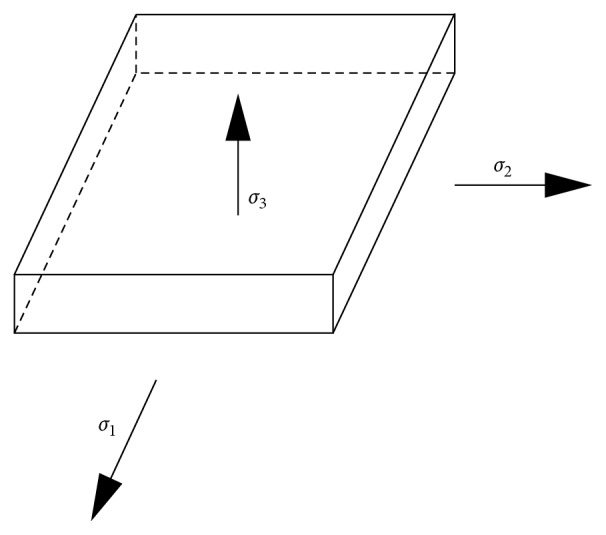
The stresses in each direction.

To apply the requirements of the mechanical performance model in the following work, the stresses in each direction of the model are supposed to be considered [16, 17] (see Figure 5).

*λ*_1_, *λ*_2_, and *λ*_3_ are the *x*, *y*, and *z* directions of the main (extension) deformation rate, respectively, and are given by

(1) $$\begin{array}{c} \lambda_{1}=\frac{x}{x_{0}},\\ \lambda_{2}=\frac{y}{y_{0}},\\ \lambda_{3}=\frac{d}{d_{0}}, \end{array}$$

where *x*, *y*, and *d* are the length, width, and thickness, respectively, and *x*_0_, *y*_0_, and *d*_0_ are the corresponding initial values before deformation.

As the material is incompressible, its volume is kept constant before and after deformation, giving

(2) $$\begin{array}{c} xy\;d=x_{0}y_{0}d_{0}. \end{array}$$

Therefore,

(3) $$\begin{array}{c} \frac{xy\;d}{x_{0}y_{0}d_{0}}=\lambda_{1}\lambda_{2}\lambda_{3}=1. \end{array}$$

The physical properties of the material are mainly subjected to *W*. Each model of the material is a special form of *W* [18, 19]. If the form of *W* is determined, the Cauchy stress tensor *P* can be given by the equation below:

(4) $$\begin{array}{c} \sigma =-pI+2\frac{\partial W}{\partial I_{1}}B-2\frac{\partial W}{\partial I_{2}}B^{-1}, \end{array}$$

where *I* is the unit tensor, which is the left Gaussian deformation tensor, and *P* is the hydrostatic pressure introduced by the incompressibility assumption.

Since *I*_i_ is invariable under any changes of *B*, the following is given:

(5) $$\begin{array}{c} \left\{\begin{array}{c} I_{1}=B,\\ I_{2}=\frac{1}{2}\left[I_{1}^{2}-t_{\text{r}}\left(B^{2}\right)\right],\\ I_{3}=\det\;B, \end{array}\right. \end{array}$$

where *B* is the component of the Green strain tensor. The relationship between the invariants and principal elongation is a function of *B*.

(6) $$\begin{array}{c} \left\{\begin{array}{c} I_{1}=t_{\text{r}}\left[B\right]=B_{ii}=\lambda_{1}^{2}+\lambda_{2}^{2}+\lambda_{3}^{2},\\ I_{2}=\frac{1}{2}{\left(t_{\text{r}}\left[B\right]\right)}^{2}-\left(t_{\text{r}}{\left[B\right]}^{2}\right)=\frac{1}{2}\left(B_{ii}B_{ii}-B_{ij}B_{ji}\right)\\ =\lambda_{1}^{2}\lambda_{2}^{2}+\lambda_{2}^{2}\lambda_{3}^{2}+\lambda_{3}^{2}\lambda_{1}^{2}=\frac{1}{\lambda_{1}^{2}}+\frac{1}{\lambda_{2}^{2}}+\frac{1}{\lambda_{3}^{2}},\\ I_{3}=\det\;B=\lambda_{1}^{2}\lambda_{2}^{2}\lambda_{3}^{2}. \end{array}\right. \end{array}$$

The isotropic and incompressible deformation process of the ILG is given by

(7) $$\begin{array}{c} \sqrt{I_{3}}=\lambda_{1}\lambda_{2}\lambda_{3}=1. \end{array}$$

According to formulas (4) and (6), it can be obtained that

(8) $$\begin{array}{c} \sigma_{i}=2\left[\lambda_{i}^{2}\frac{\partial W}{\partial I_{1}}-\frac{1}{\lambda_{i}^{2}}\frac{\partial W}{\partial I_{2}}\right]-p, \end{array}$$

where *I*_1_, *I*_2_, and *I*_3_ are the relative changes in the length, surface area, and volume of the elastomer, respectively.

### 4.2. Ogden Model

According to a comprehensive comparison of various hyperelastic constitutive models, the Ogden model is selected in this work. The Ogden model is a preferred energy function in finite element simulation analysis. In this paper, the mechanical properties of ILG-incompressible hyperelastic materials are described by the Ogden model. The mechanical properties of the ionic gel materials were then studied using the Ogden formula to describe the nonlinearity of the ionic gels [20, 21].

The strain energy function of the Ogden model equation is given as follows:

(9) $$\begin{array}{c} W=\overset{N}{\underset{i=1}{\sum }}\frac{\mu_{i}}{\alpha_{i}}\left(\lambda_{1}^{\alpha_{i}}+\lambda_{2}^{\alpha_{i}}+\lambda_{3}^{\alpha_{i}}-3\right),\;\left(1\leq N\leq 3\right), \end{array}$$

where *μ*_*i*_ and *α*_*i*_ are material constants.

The form of the strain energy potential is given by

(10) $$\begin{array}{c} W=\frac{\mu_{1}}{\alpha_{1}}\left(\lambda_{1}^{\alpha_{1}}+\lambda_{2}^{\alpha_{1}}+\lambda_{3}^{\alpha_{1}}-3\right)+\frac{\mu_{2}}{\alpha_{2}}\left(\lambda_{1}^{\alpha_{2}}+\lambda_{2}^{\alpha_{2}}+\lambda_{3}^{\alpha_{2}}-3\right)+\frac{\mu_{3}}{\alpha_{3}}\left(\lambda_{1}^{\alpha_{3}}+\lambda_{2}^{\alpha_{3}}+\lambda_{3}^{\alpha_{3}}-3\right),\;\left(N=3\right). \end{array}$$

Combining equation (8) with equation (9), it can be obtained that

(11) $$\begin{array}{c} \sigma_{i}=\lambda_{i}\frac{\partial W}{\partial \lambda_{i}}-p. \end{array}$$

Substituting equation (9) into equation (11) gives

(12) $$\begin{array}{c} \sigma_{i}=\overset{3}{\underset{k=1}{\sum }}\mu_{k}\lambda_{i}^{\alpha_{k}}-p. \end{array}$$

The stresses in each direction are given by

(13) $$\begin{array}{c} \sigma_{1}=\mu_{1}\lambda_{1}^{\alpha_{1}}+\mu_{2}\lambda_{1}^{\alpha_{2}}+\mu_{3}\lambda_{1}^{\alpha_{3}}-p, \end{array}$$

(14) $$\begin{array}{c} \sigma_{2}=\mu_{1}\lambda_{2}^{\alpha_{1}}+\mu_{2}\lambda_{2}^{\alpha_{2}}+\mu_{3}\lambda_{2}^{\alpha_{3}}-p, \end{array}$$

(15) $$\begin{array}{c} \sigma_{3}=\mu_{1}\lambda_{3}^{\alpha_{1}}+\mu_{2}\lambda_{3}^{\alpha_{2}}+\mu_{3}\lambda_{3}^{\alpha_{3}}-p. \end{array}$$

When the ILG is uniformly stretched in the *x* direction, *λ*_2_=*λ*_3_, which can be calculated with equation (3), giving

(16) $$\begin{array}{c} \lambda_{2}=\lambda_{3}=\frac{1}{\sqrt{\lambda_{1}}}. \end{array}$$

Because only the axial tensile deformation is considered, the stresses in the other two directions are zero.

(17) $$\begin{array}{c} \sigma_{2}=\sigma_{3}=0. \end{array}$$

Combining equation (14) or (15) with equation (17), it can be obtained that

(18) $$\begin{array}{c} p=\mu_{1}\lambda_{2}^{\alpha_{1}}+\mu_{2}\lambda_{2}^{\alpha_{2}}+\mu_{3}\lambda_{2}^{\alpha_{3}}=\mu_{1}\lambda_{3}^{\alpha_{1}}+\mu_{2}\lambda_{3}^{\alpha_{2}}+\mu_{3}\lambda_{3}^{\alpha_{3}}. \end{array}$$

Therefore,

(19) $$\begin{array}{c} \sigma_{1}=\mu_{1}\lambda_{1}^{\alpha_{1}}+\mu_{2}\lambda_{1}^{\alpha_{2}}+\mu_{3}\lambda_{1}^{\alpha_{3}}-\left(\mu_{1}\lambda_{2}^{\alpha_{1}}+\mu_{2}\lambda_{2}^{\alpha_{2}}+\mu_{3}\lambda_{2}^{\alpha_{3}}\right)\\ =\mu_{1}\left(\lambda_{1}^{\alpha_{1}}-\lambda_{2}^{\alpha_{1}}\right)+\mu_{2}\left(\lambda_{1}^{\alpha_{2}}-\lambda_{2}^{\alpha_{2}}\right)+\mu_{3}\left(\lambda_{1}^{\alpha_{3}}-\lambda_{2}^{\alpha_{3}}\right)\\ =\mu_{1}\left(\lambda_{1}^{\alpha_{1}}-\lambda_{1}^{-\left(1/2\right)\alpha_{1}}\right)+\mu_{2}\left(\lambda_{1}^{\alpha_{2}}-\lambda_{1}^{-\left(1/2\right)\alpha_{2}}\right)+\mu_{3}\left(\lambda_{1}^{\alpha_{3}}-\lambda_{1}^{-\left(1/2\right)\alpha_{3}}\right). \end{array}$$

## 5. Equivalent Circuit Model

The impedance of the ionized EAP actuator is capacitive at low frequencies and resistive at high frequencies. Almost all of the equivalent circuit models are therefore composed of capacitors and resistive elements.

The driving current of the ionic liquid polymer gel actuator can be considered as a combination of the ion current, displacement current, and electron current. The ion current of the ionic polymer-metal composite (IPMC) is formed by the directional movement of the hydrated cations, and the ion current of the ionic liquid polymer gel actuator is formed by the simultaneous movement of the anions and cations in opposite directions [22, 23].

The current response of the ILG actuator is mainly composed of the ion current and the electron current, the dynamic performance is exhibited by the ion current characteristics, and the static performance is exhibited by the electron current characteristics.

**Figure 6:**
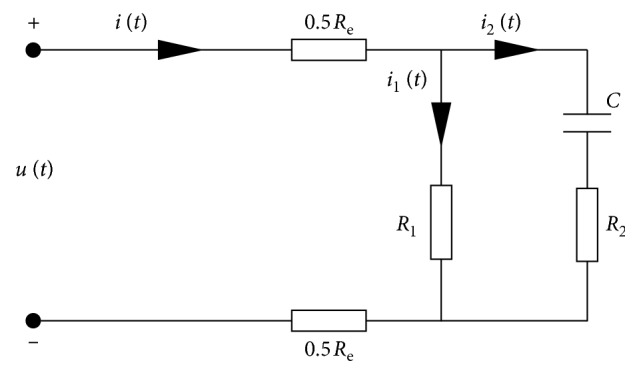
Equivalent circuit model.

The simplified resistance-capacitance (*R*-*C*) equivalent circuit model of the ILG flexible actuator is illustrated in Figure 6. *R*_1_ is the direct current (DC) equivalent resistance of the actuator, and this branch represents the electron current branch, reflecting the static response of the static characteristics. *R*_2_ is the alternating current (AC) equivalent resistance of the actuator, *C* is the equivalent capacitor of the actuator, and this branch represents the ion current branch, reflecting the dynamic characteristics of the drive. *R*_o_ is the surface electrode resistance of the actuator. Since the electron current response of the ILG actuator is much faster than the ion current response and the initial value is relatively small, the nonlinearity of the electron current branch is thus negligible in the equivalent circuit. The initial energy storage of the dynamic components in the circuit is assumed to be zero, and the initial voltages at both ends of the capacitor are therefore equal to zero.

(20) $$\begin{array}{c} \left\{\begin{array}{c} i_{1}\left(t\right)=\frac{u_{\text{RC}}\left(t\right)}{R_{1}},\\ R_{2}i_{2}\left(t\right)+\frac{1}{C}\int i_{2}\left(t\right)\;dt=u_{\text{RC}}\left(t\right),\\ i\left(t\right)=i_{1}\left(t\right)+i_{2}\left(t\right),\\ u_{\text{RC}}\left(t\right)+i\left(t\right)R_{e}=u\left(t\right), \end{array}\right. \end{array}$$

where *i*_1_ is the electron current of the actuator, *i*_2_ is the ion current of the actuator, *i* is the input current of the actuator, *u* is the input voltage, and *u*_RC_ is the actuator actuation voltage.

Under zero-input conditions, the transfer function of the input current and input voltage of the actuator can be obtained from the Laplace transform of equation (20), giving

(21) $$\begin{array}{c} \frac{I\left(s\right)}{U\left(s\right)}=\frac{\left(R_{1}+R_{1}\right)Cs+1}{\left[R_{1}R_{2}+R_{e}\left(R_{1}+R_{2}\right)\right]Cs+R_{1}+R_{e}}. \end{array}$$

When the input is a step signal with an amplitude of *u*_0_, substituting in equation (21) gives

(22) $$\begin{array}{c} I\left(s\right)=\frac{\left(R_{1}+R_{1}\right)Cs+1}{\left[R_{1}R_{2}+R_{e}\left(R_{1}+R_{2}\right)\right]Cs+R_{1}+R_{e}}\frac{U_{0}}{s}=\frac{a_{0}}{s}+\frac{a_{1}}{s+\lambda }, \end{array}$$

(23) $$\begin{array}{c} \left\{\begin{array}{c} a_{0}=U_{0}\frac{R_{1}R_{2}+R_{1}R_{e}+R_{2}R_{2}}{\left[R_{1}R_{2}+R_{e}\left(R_{1}+R_{2}\right)\right]\left(R_{1}+R_{e}\right)},\\ a_{1}=U_{0}\frac{R_{1}^{2}}{\left[R_{1}R_{2}+R_{e}\left(R_{1}+R_{2}\right)\right]\left(R_{1}+R_{e}\right)},\\ \lambda =\frac{R_{1}+R_{e}}{\left[R_{1}R_{2}+R_{e}\left(R_{1}+R_{2}\right)\right]C}. \end{array}\right. \end{array}$$

The current time-domain response of the equivalent circuit model can be obtained from the inverse Laplace transform of equation (22), giving

(24) $$\begin{array}{c} i\left(t\right)=a_{0}+a_{1}e^{-\lambda t}. \end{array}$$

For the equivalent circuit model, the fitting precision function is given by

(25) $$\begin{array}{c} J_{1}={\left\|i_{\text{s}}-i_{\text{e}}\right\|}_{2}^{2}=\overset{n}{\underset{j=1}{\sum }}{\left(i_{\text{s}j}-i_{\text{e}j}\right)}^{2}, \end{array}$$

where *i*_s_ is the current time-domain response of the equivalent circuit model and *i*_e_ is the experimental current data.

The system parameters are identified by the step-response curve and least-squares method. The optimal parameters are *a*_0_=0.0060, *a*_1_=0.2058, and *λ*=0.1470.

Therefore,

(26) $$\begin{array}{c} i\left(t\right)=0.006+0.2058e^{-0.147t}. \end{array}$$

## 6. CPG Control

The CPG is an oscillation unit composed of a series of intermediate neurons, and the entire CPG control network is a complex distributed neural network, which combines a neural oscillator and a multiple-reflection feedback loop system. The CPG network can generate a stable phase interlocking relationship by mutual inhibition of the neurons and can also generate rhythmic motions by self-oscillating excitation of body-related parts. Each cell (motor neuron) is assumed to activate a single actuator in the motor system; the number of cells of the CPG is thus equal to the number of actuators for a structure with *n* actuators. The synaptic connections among the neurons in the CPG are elastic, and the CPG network can therefore provide a variety of output modes and control the animals to achieve different movement patterns [24–26].

**Figure 7:**
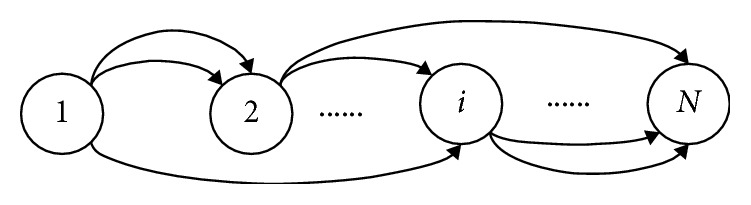
Chained CPG network topology.

Each oscillator in the CPG chain is aligned in a chain shape, and the rhythmic signal of a body fluctuation can be transmitted from one end to the other; the constant phase lag between every two segments is fixed so that the animal maintains a body-long wavelength at any speed, as shown in Figure 7. When an earthworm creates fluctuating movement, traveling waves propagate from the tail to the head, leading to continuous bending of the muscles of the segment from the tail to the head. To implement this kind of movement mode, a traveling wave propagating from the cellular neural network cell associated with the last (tail) segment to the cell associated with the first (head) segment is supposed to be generated.

Generally, a degree of freedom of movement is subjected to an oscillator, and a plurality of oscillators can form different topologies to control the coordinated motion of the animal.

### 6.1. Structure of the Actuator Model

A joint arm has a limited number of degrees of freedom, and an ILG soft robot actuator is a continuum with an unlimited number of degrees of freedom. Therefore, the control of soft robots is a great challenge. A discrete substitution of the continuous description of the soft robot actuator is therefore used to simplify the model.

The soft robot actuator is thus modeled using the point quality and the spring of the 2D array. The masses of all the soft robot actuators are concentrated on the discrete points connected by a massless damping spring. In this paper, the soft actuator model is 2D, and all force vectors are in the *x*-*y* plane, where *y* is the gravitational direction, and the motion of the soft robot is limited in the plane.

1. The simulated motion of the model is free motion, without interaction with any other objects.
2. The soft robot actuator is discretized into 15 segments. A comparison of the accuracy of the 15-segment model and that of the 30-segment model shows that the former can provide the desired continuous structure of a soft robot actuator.
3. All the forces contained in the model are limited in the *x*-*y* plane. When this model is compared with the 3D model, although the generality is limited, the computational cost is greatly reduced, and the reaching movement of the soft robot is also achievable.

**Figure 8:**
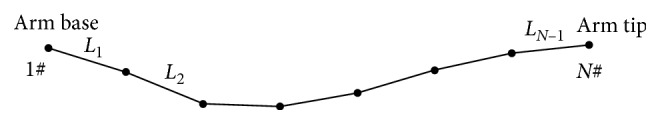
The actuator model.

In this paper, there are 15 segments in this model.  Figure 8 shows the general structure of the modeled soft robot actuator.

The bending deformation of the ILG is a result of the differences in volume caused by the movement of the cations and anions in opposite directions. The reliability of the soft robot actuator model is based on the assumption that the ILG is incompressible. Due to the constant-volume constraints, the bending deformation of the soft robot actuator will shorten the length of one side while increasing the length of the other side. A simple physical mechanism is used in the 2D model, in which the motion of the soft robot actuator is almost unrestricted in the plane, and the force is transferred from one side of the soft robot actuator to the other side, without the need for a rigid skeleton.

### 6.2. Dynamic Model of Soft Robot Actuator

The soft robot actuator model built in this work is a 2D model in which all forces are vectors in the *x*-*y* plane. The movement of the soft robot actuator is thus limited to the plane. In this paper, three types of forces for the actuator are involved. The simplified motion equation can be given by

(27) $$\begin{array}{c} M\ddot{q}=F_{\text{m}}+F_{\text{g}}+F_{\text{c}}, \end{array}$$

where *M* is the diagonal mass matrix, *q* is the position vector, *F*_m_ is the internal force generated by the ILG, *F*_g_ is the vertical force resulting from the influences of gravity, and *F*_c_ is the internal force that maintains constant-volume constraints.

The equation is numerically integrated using an explicit Runge–Kutta equation with an adaptive step size. The initial conditions are the initial positions of all discrete mass points. The initial speed is set to zero.

The internal force of the actuator is simulated with an ideal damping spring and is caused by a change in the applied spring constant. The spring constant is adjusted to allow the user to control the movement of the actuator. The relevant physical theoretical formula is then utilized to calculate the gravity and traction of the soft robot actuator.

As a result of discretization, every linear segment of the actuator in the model exerts a force as below [27], giving

(28) $$\begin{array}{c} f\left(t\right)=\left[k_{0}+k_{\max}a\left(t\right)\right]\left[l\left(t\right)-l_{\text{rest}}\right]+\alpha \frac{dl\left(t\right)}{dt}, \end{array}$$

where *l*_rest_ is the rest length of the soft robot actuator and is selected as the maximum length where both active and passive forces in a real actuator are zero; the linear damping coefficient *α* has dimensions of Ns/m; *k*_0_ is the passive spring constant of the actuator and *k*_max_ is the maximum active spring constant of the actuator, both of which have dimensions of N/m; and *a*(*t*) is a dimensionless activation function.

### 6.3. CPG Network Model

The CPG network interacts with the environment and controls robot joint signals with environmental adaptability. An actuator with N segments can control the motion of the soft robot using N oscillators, from the tail to the head of the drive, consequently causing the ILG actuator to bend from the tail to the head. The Kimura neuron oscillator consists of two mutually suppressed neurons, and each joint of the robot is driven by a neural oscillator [28, 29]. The output of the two neurons is subtracted as the output of the oscillator, and the mathematical model is summarized as the equations below:

(29) $$\begin{array}{c} \left\{\begin{array}{c} T_{\text{r}}{\dot{u}}_{\left\{\text{e,f}\right\}i}+u_{\left\{\text{e,f}\right\}i}=w_{\text{fe}}y_{\left\{\text{e,f}\right\}i}-\beta v_{\left\{\text{e,f}\right\}i}+\overset{n}{\underset{j=1}{\sum }}w_{ij}y_{\left\{\text{e,f}\right\}i}+s_{0},\\ T_{\text{a}}{\dot{v}}_{\left\{\text{e,f}\right\}i}+v_{\left\{\text{e,f}\right\}i}=y_{\left\{\text{e,f}\right\}i},\\ y_{\left\{\text{e,f}\right\}i}=\max\left(0,u_{\left\{\text{e,f}\right\}i}\right),\\ y_{i}=-y_{\left\{\text{e}\right\}i}+y_{\left\{\text{f}\right\}i}, \end{array}\right. \end{array}$$

where *i*, e, and f represent the flexor and extensor neurons of the *i* ^th^ CPG unit, respectively, *u*_{e,f}_ is the internal state of the neuron, *v*_{e,f}_ is the self-inhibitory state of the neuron, and *y*_{e,f}_ is the output of the neuron. *T*_r_ and *T*_a_ represent the rise time and adaptation time constant, respectively. *w*_fe_ represents the mutual inhibition coefficient of neurons, *β* represents the self-suppression coefficient of neurons, and *s*_0_ represents the tonic input and determines the amplitude of the CPG output.

The CPG network is used to coordinate the multidegree of freedom of the control robot, and the robot motion mode can be adjusted by changing the parameters of the CPG. A mathematical model of each segment of the actuator can be obtained after discretizing the continuous soft robot actuator.

The CPG model of the soft actuator can be described as follows:

(30) $$\begin{array}{c} \left\{\begin{array}{c} T_{\text{r}}{\dot{u}}_{1}+u_{1}=-\beta v_{1}-w\max\left(0,u_{2}\right)+s_{0},\\ T_{\text{a}}{\dot{v}}_{1}+uv_{1}=\max\left(0,u_{1}\right),\\ T_{\text{r}}{\dot{u}}_{2}+u_{2}=-\beta v_{2}-w\max\left(0,u_{1}\right)-w\max\left(0,u_{3}\right)+s_{0},\\ T_{\text{a}}{\dot{v}}_{2}+uv_{2}=\max\left(0,u_{2}\right),\\ \cdots \\ T_{\text{r}}{\dot{u}}_{15}+u_{15}=-\beta v_{15}-w\max\left(0,u_{14}\right)+s_{0},\\ T_{\text{a}}{\dot{v}}_{15}+uv_{15}=\max\left(0,u_{15}\right),\\ y_{1}=\max\left(0,u_{1}\right)-\max\left(0,u_{2}\right),\\ y_{2}=\max\left(0,u_{2}\right)-\max\left(0,u_{3}\right),\\ \cdots \\ y_{14}=\max\left(0,u_{14}\right)-\max\left(0,u_{15}\right), \end{array}\right. \end{array}$$

where *u*_1_, *u*_2_, and *u*_3_ represent the membrane potentials, *v*_1_, *v*_2_, and *v*_3_ represent the adaptation or fatigue properties in real neurons, and *y*_1_, *y*_2_, and *y*_3_ represent the output signals of the CPG.

**Figure 9:**
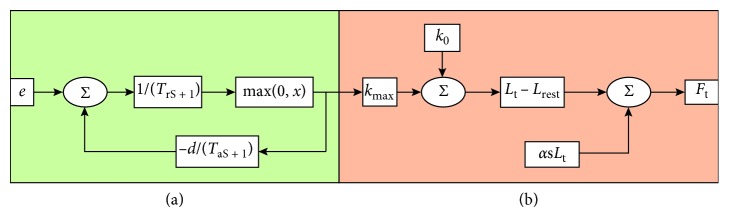
(a) A model of interaction between the soft robot actuator and the CPG (in green). (b) The interaction between the soft robot actuator and the CPG (in amber).

Yekutieli et al. [30] demonstrated that the mechanism of bending propagation is an internal force enhancement wave. It is therefore reasonable to add the CPG single-cycle output of the active wave processing to the soft actuator. The interaction between the soft actuator and the CPG is shown by the diagram in Figure 9.

In Figure 9, the left panel is a control block diagram of the CPG, and the right panel is a control block diagram of the soft actuator with the Laplace transform [31].

**Figure 10:**
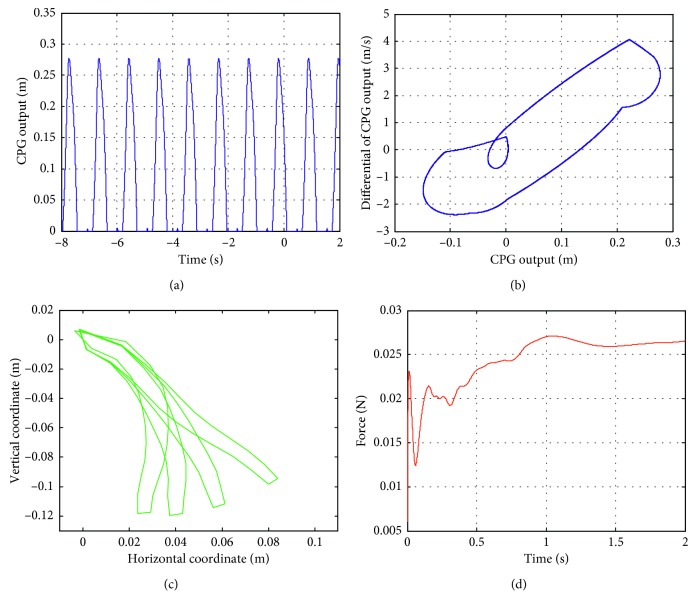
(a) CPG outputs. (b) Phase diagrams of these CPGs. (c) A sequence of the soft robot actuator movements. (d) The internal force of the first segment.

The basic values of the CPG parameters are set as *T*_r_ = 0.12 s, *T*_a_ = 0.3 s, *d* = 3, *w*=3, and *e* = 1. When the initial input value is set to [0.12 0 0 0 0 0.12], the output and phase diagrams of the CPG are obtained, as plotted in Figures 10(a) and 10(b), respectively. The motion trajectories of the soft actuator for the durations of 0.5 s, 0.7 s, 1.0 s, and 1.5 s are selected, and the motion path of the soft actuator is plotted in Figure 10(c). The internal force of the soft actuator is also obtained, as shown in Figure 10(d). The motion in Figure 10(c) can be used to simulate the soft actuator motion. Since the internal force of each segment of the soft actuator is similar to that of the first section, the internal force of the first section is shown in Figure 10(d) only.

### 6.4. Simulation Analysis

#### 6.4.1. The Effects of Various *e* Values on the Soft Actuator Motion

**Figure 11:**
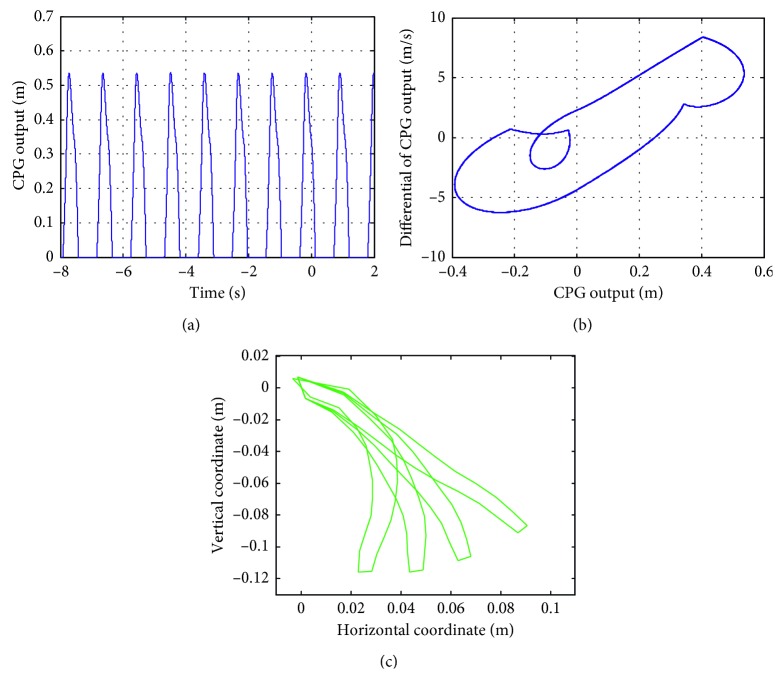
Output and phase diagrams of the CPG and the soft actuator trajectory with *e* = 2.

In the simulation, the value of *e* increases with an increment of 1 in the range of (0, 50). When *e* = (0, 4), the output and phase curves of the CPG are obtained, as shown in Figures 11(a) and 11(b), respectively, where the shape of the phase diagram of the CPG is a limit cycle. The motion of the soft actuator can also be simulated. The motions of a series of soft actuators are shown in Figure 11(c), and it can be seen that the soft actuators reach the specified location in a smooth way.

#### 6.4.2. The Effects of Various *T*_r_ Values on the Soft Actuator Motion

**Figure 12:**
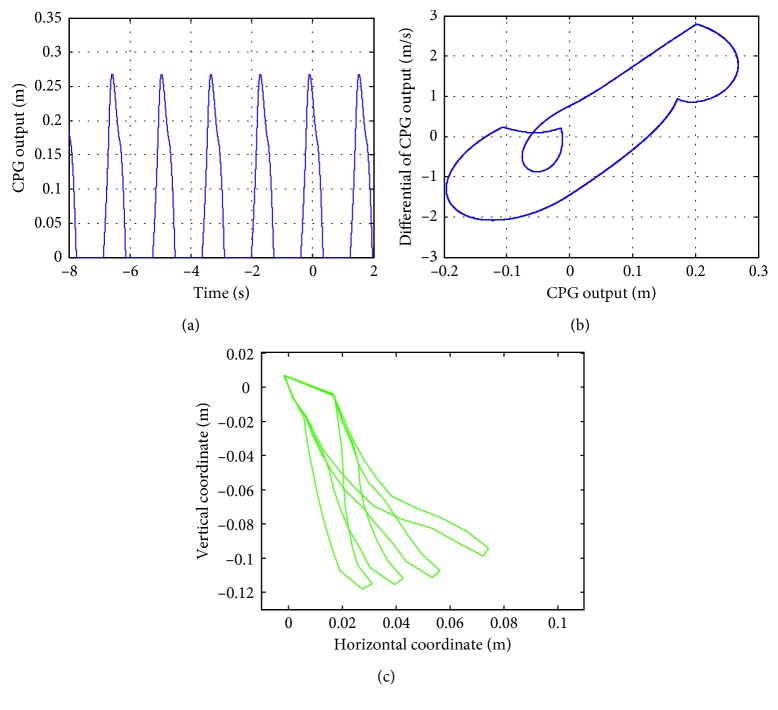
Output and phase diagrams of CPG and the soft actuator trajectory with *T*_r_ = 0.15.

When *e* = 1, the value of *T*_r_ increases with an increment of 0.1 in the range of (0, 1). When *T*_r_ ∈ (0,0.3), the output and phase curves of the CPG are obtained, as shown in Figures 12(a) and 12(b), respectively, where the shape of the phase diagram of the CPG is a limit cycle. The motion of the soft actuator can also be simulated. The motions of a series of soft actuators are shown in Figure 12(c), and it can be seen that the soft actuators reach the specified location in a smooth way.

#### 6.4.3. The Effects of *d*  and  *w* on the Soft Actuator Motion

**Figure 13:**
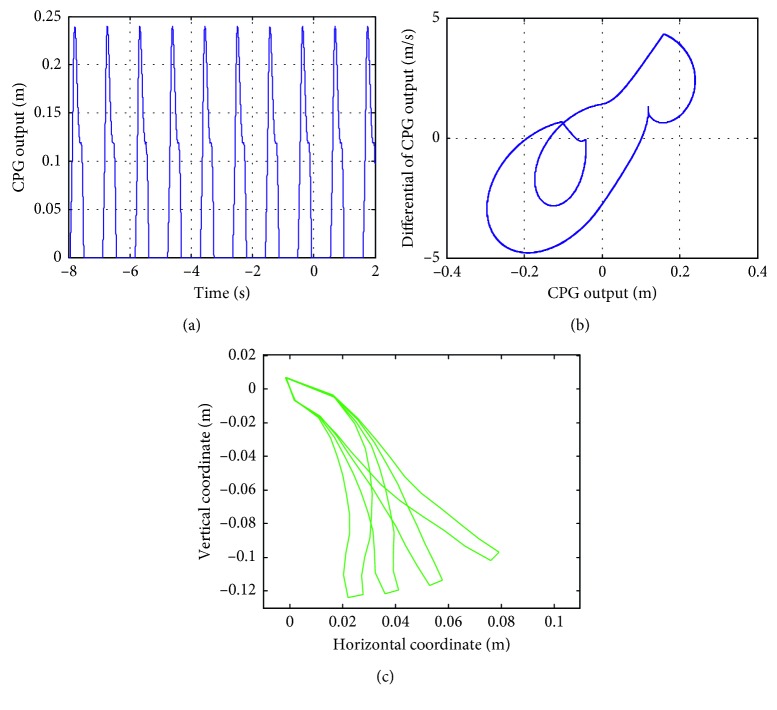
Output and phase diagrams of the CPG and the soft actuator trajectory with *d* = *w* = 6.

The effects of various *d*  and  *w* values on the soft actuator when *e* = 1 and *T*_r_ = 0.1 are investigated. The values of *d*  and  *w* increase with an increment of 1 in the range of (0, 50). When *d*=*w* ∈ (3,12), the output and phase curves of the CPG are obtained, as shown in Figures 13(a) and 13(b). The movement of the soft actuator can also be simulated, as shown in Figure 13(c), and it can be seen that the soft actuators reach the designated location in a smooth way.

In summary, a larger amplitude causes the soft actuator to become disordered. A smaller frequency destroys the limit cycle, causing failure of the maintenance of the soft actuator movement. The parameters *d*  and  *w* also affect the shape of the CPG phase curve of the soft actuator.

## 7. Conclusion

This paper demonstrates a new soft actuator design and includes information on the structure of the soft actuator, the material composition, the material mechanical properties analysis, and the CPG control simulation analysis. The structure of the soft actuator is introduced in detail, which introduces a new direction for the development of soft actuators. The material composition of the soft actuator is described, and its mechanical properties are analyzed using a hyperelastic model. The principle of bending deformation of the soft actuator is based on the fact that the volume of the cation side is much larger than the volume of the anion side and that the volume on the cathode side of the actuator expands, causing the volume of the anode side to shrink; thus, the actuator bends toward the anode side. The CPG control is demonstrated in detail, and the motion of the soft actuator under various conditions is simulated. By adjusting the parameters of the CPG to achieve the motion of the soft actuator, the actuator can reach any specified position.

A soft actuator behaves similarly to octopus muscles, without any stiff skeletal support. The biomechanical properties of an octopus arm make it possible to perform tasks without being a skeletal arm. Under the driving force of an applied voltage, continuous bending deformation occurs, which increases the contact area between an actuator and an object. Additionally, this bending deformation reduces damage to objects, such as eggs, when they are being grabbed. The simulation results will help researchers to further understand the motion of soft actuators and will contribute to their development. Due to the chemical stability, thermal stability, and simple ion transport of the ILG, it is an advantageous choice for soft actuator materials. ILGs offer low weight and high distortion and can be controlled by low-voltage signals; thus, ILGs are suitable for the production of soft robot actuators. A soft actuator with a real-time learning control mechanism will thus produce highly versatile applications, which is also the direction of future related work.

## Data Availability

The data used to support the findings of this study are included within the article.
